# Diagnostic role of circulating extracellular matrix-related proteins in non-small cell lung cancer

**DOI:** 10.1186/s12885-018-4772-0

**Published:** 2018-09-18

**Authors:** Francesca Andriani, Elena Landoni, Mavis Mensah, Federica Facchinetti, Rosalba Miceli, Elda Tagliabue, Marta Giussani, Maurizio Callari, Loris De Cecco, Mario Paolo Colombo, Luca Roz, Ugo Pastorino, Gabriella Sozzi

**Affiliations:** 1 Department of Research, Tumor Genomics Unit, Fondazione IRCCS Istituto Nazionale dei Tumori, Via Venezian 1, 20133 Milan, Italy; 2 Department of Applied Research and Technical Development, Medical Statistics and Biometry Unit, Fondazione IRCCS Istituto Nazionale dei Tumori, Milan, Italy; 3 Department of Research, Molecular Targeting Unit, Fondazione IRCCS Istituto Nazionale dei Tumori, Milan, Italy; 4 Department of Applied Research and Technical Development, Biomarkers, Fondazione IRCCS Istituto Nazionale dei Tumori, Milan, Italy; 5 Present address: Cancer Research UK Cambridge Institute, University of Cambridge, Cambridge, UK; 6 Department of Applied Research and technical Development, Genomics, Fondazione IRCCS Istituto Nazionale dei Tumori, Milan, Italy; 7 Department of Research, Molecular Immunology Unit, Fondazione IRCCS Istituto Nazionale dei Tumori, 20133 Milan, Italy; 8 Department of Surgery, Thoracic Surgery Unit Fondazione IRCCS Istituto Nazionale dei Tumori, Milan, Italy

**Keywords:** Lung cancer, Extracellular matrix (ECM), Circulating biomarkers, SPARC, Secreted protein acidic and rich in cysteine, CAF, Cancer associated fibroblasts

## Abstract

**Background:**

Interactions between cancer cells and the surrounding microenvironment are crucial determinants of cancer progression. During this process, bi-directional communication among tumor cells and cancer associated fibroblasts (CAF) regulate extracellular matrix (ECM) deposition and remodeling. As a result of this dynamic process, soluble ECM proteins can be released into the bloodstream and may represent novel circulating biomarkers useful for cancer diagnosis. The aim of the present study was to measure the levels of three circulating ECM related proteins (COL11A1, COL10A1 and SPARC) in plasma samples of lung cancer patients and in healthy heavy-smokers controls and test whether such measurements have diagnostic or prognostic value.

**Methods:**

Gene expression profiling of lung fibroblasts isolated from paired normal and cancer tissue of NSCLC patients was performed by gene expression microarrays. The prioritization of the candidates for the study of circulating proteins in plasma was based on the most differentially expressed genes in cancer associated fibroblasts. Soluble ECM proteins were assessed by western blot in the conditioned medium of lung fibroblasts and by ELISA assays in plasma samples.

**Results:**

Plasma samples from lung cancer patients and healthy heavy-smokers controls were tested for levels of COL11A1 and COL10A1 (*n* = 57 each) and SPARC (*n* = 90 each). Higher plasma levels of COL10A1 were detected in patients (*p* ≤ 0.001), a difference that was driven specifically by females (*p* < 0.001). No difference in COL11A1 levels between patients and controls was found. SPARC levels were also higher in plasma patients than controls (*p* < 0.001) with good performance in discriminating the two groups (AUC = 0.744). No significant association was observed between plasma proteins levels and clinicopathological features or survival.

**Conclusion:**

Soluble factors related to proficient tumor-stroma cross-talk are detectable in plasma of primary lung cancer patients and may represent a valuable complementary diagnostic tool to discriminate lung cancer patients from healthy heavy-smokers individuals as shown for the SPARC protein.

**Electronic supplementary material:**

The online version of this article (10.1186/s12885-018-4772-0) contains supplementary material, which is available to authorized users.

## Background

Lung cancer represents a major health issue worldwide due to its high incidence and mortality rates [1]. The disease is often diagnosed at advanced stages when current clinical options are largely ineffective resulting five years survival rates of less than 10% [2]. Identification of circulating molecular biomarkers is therefore critical to improve early detection of lung cancer and represents an important approach with a large clinical potential [3]. Despite the recent improvements in the characterization of different circulating biomarkers such as cell free DNA [4, 5], circulating tumor cells [6–8], extracellular vesicles or circulating miRNA [9, 10], the search for optimal biomarkers still remains a challenge. In the past, most studies were centered on identifying potential biomarkers using molecules differentially expressed by tumor cells. However, in the last few years, the concept that each tumor is a complex system composed by both, cancer cells and the surrounding stroma, represented by a variety of cell types such as fibroblasts, immune and endothelial has been well established [11]. This notion has important implications for biomarkers research as novel candidates with diagnostic or prognostic value can potentially be obtained by analyzing molecules produced by stromal cells during their interactions with cancer cells [12, 13].

In particular, activated fibroblasts play a prominent role in lung carcinogenesis due to their abilities to trigger several signaling pathways implicated in tumor formation and metastasis [14–16]. It has also been demonstrated that cancer-associated fibroblasts (CAFs) within the reactive stroma are responsible for deposition of elevated amounts of extracellular matrix (ECM) [17]. In physiological condition, ECM provides mechanical and biochemical support to the surrounding cells and is actively involved in cell proliferation and migration [18]. On the other hand, under pathological conditions the interactions between activated fibroblasts and epithelial cells result in production of different growth factors, cytokines and proteases which modify the surrounding ECM, by changing its composition and facilitating pathological alterations such as chronic obstructive pulmonary disease (COPD) or idiopathic pulmonary fibrosis (IPF) [19] and potentially leading to cancer development [20, 21].

As a consequence of this remodeling, proteins related to ECM are released into blood and could be considered as potential novel circulating biomarkers [22]. Therefore, new biomarkers for early detection of lung cancer derived from remodeling of ECM could be developed by identifying candidate genes and pathways from gene expression profiling of cancer associated fibroblasts (CAF). A recent review started to unravel the key pathways involved in their functional effects highlighting the existence of common mechanisms as well as specificities in different cancer types (breast, prostate and lung cancer) [23]. The results showed that most of the commonly enriched gene sets characterizing tumor-promoting fibroblasts were related to structural ECM molecules and ECM organization (e.g. collagens) and in particular, to the ECM3 signature, an ECM-based signature already found associated with bad prognosis in aggressive breast carcinomas (i.e. grade III) [24]. Mechanistically the deposition of collagens in the surrounding tumor influences cancer cells behavior promoting cancer progression and invasion in several cancer types [25, 26]. However, the utility of any specific collagen fragment as plasma circulating biomarker in lung cancer remains unproven. Several studies have also pointed out the importance of another lung microenvironment-related protein, secreted protein acidic and rich in cysteine (SPARC), a collagen-binding matricellular protein considered as a key player in the tumor progression most likely by supporting crosstalk at the tumor–stroma interface [27, 28]. SPARC has been predominantly detected in the tumor-associated stroma, specifically in ECM produced by activated fibroblasts. Interestingly, the localization of SPARC in NSCLC tissues is linked to disease prognosis. In fact, high levels of SPARC expression within NSCLC tumor tissues, are associated to longer survival, while its absence represents a negative prognostic factor. On the other hand, high expression of SPARC in the stroma is associated with poor overall survival in lung cancer patients [29]. Since changes in the ECM can occur early in cancer progression, in this study we aimed to identify plasma circulating proteins originated by the ECM compartment and to investigate their potential utility as biomarkers for early diagnosis of lung cancer. In addition, we aimed to explore the association of ECM proteins with different clinical parameters and their potential prognostic value.

## Methods

### Patient characteristics and tissue sampling

The current study was approved by the Fondazione IRCCS, Istituto Nazionale dei Tumori Ethics Review Board and included all consecutive patients from whom plasma samples were available and who underwent a complete anatomical resection for primary lung cancer at the Thoracic Surgery Division of the National Cancer Institute of Milan, from January 2012 to July 2014. Healthy heavy smoker controls were enrolled in a lung cancer screening program (clinicaltrial.gov NCT 02247453, www.biomild.org) from January 2013 to January 2016. Written informed consent was obtained from all patients and healthy heavy smokers controls for blood collection. All cases used in this study were confirmed to be primary lung cancer by pathology review. Study participants were mainly heavy smokers (12 non-smokers for the analysis of COL11A1 and COL10A1) and were matched 1:1 to the patient cohorts according to sex and age classes (< 50, 50–54, 55–59, 60–64, 65–69, 70–75, > 75) for the analysis of COL11A1 and COL10A1 and according to sex, smoking history and age classes for the analysis of SPARC. Overall survival was the study outcome of interest, thus patients contributed with their time interval from surgery until the date of death or until 16th January 2017 for survivors. Blood collection was performed shortly before surgery to avoid the impact of surgery in the markers quantification. Plasma extraction was described elsewhere [30]. Briefly, whole blood samples (5–10 ml) were collected as first blood with spray-coated K_2_EDTA tubes (BD-Becton, Dickinson and Company, Plymouth, UK). Within 2 h, plasma was separated by a first centrifugation step at 2500 RPM at 4 °C for 10 min. The supernatant containing plasma was carefully collected avoiding the fraction closest to the lymphocytic ring. Plasma was then centrifuged a second time at 2500 RPM at 4 °C for 10 min. and the supernatant collected and stored at − 80 C until further.

### Establishment of cell cultures and conditioned medium

Cultures of primary cancer-associated (CAF) and fibroblasts derived from normal counterpart of (NF) lung cancer patients were isolated from surgical specimens and cultured as already described [31]. All cell lines were routinely tested to exclude presence of mycoplasma contamination, grown as adherent monolayer and harvested at controlled density. To obtain conditioned medium (CM), cells were grown in controlled conditions in serum free medium at the same density (cells number = 1X10^6^). After 24 h, the CM was collected, centrifuged to eliminate cell debris and stored at − 80 C until further.

### RNA purification, microarray and data analysis

Total RNA was extracted from fibroblasts cell cultures using RNA easy kit (Qiagen), followed by a clean-up treatment to remove genomic DNA. RNA purity was assessed with bioanalyzer (Agilent technologies) and concentration of RNA was evaluated by nanodrop 2000c (Thermo Scientific). Each microarray experiment was performed using 300 ng of total RNA. Procedures included first strand synthesis, second strand synthesis, double-strand cDNA clean up, in vitro transcription, cRNA purification and fragmentation. One microgram of biotinylated cRNA were finally applied to each hybridization array, Illumina Human HT-12v4 Expression BeadChip (Illumina, Inc., San Diego, CA, USA) at 58 °C for 18 h. Illumina BeadStudio software version 3.8 was used to obtain the raw data. Class comparison analysis was performed using the *limma* Bioconductor package [32]. Cancer associated fibroblast were compared with normal fibroblasts and all genes were ranked according to the modified *t-*statistics values obtained. These ranked gene lists were subjected to a Gene Set Enrichment Analysis (GSEA, v.4.0) to identify Gene Ontology terms or Canonical Pathways (BIOCARTA, KEGG, REACTOME) significantly enriched. Enrichment was considered significant at *p*-value < 0.05.

### Cell lysates, extracellular matrix and conditioned medium preparation

For cell lysates preparation, cell lines were solubilized for 1 h on ice with TNTG lysis buffer containing 50 mM Tris-HCl pH 7.5, 150 mM NaCl, 100 mM NaF, 10 mM sodium pyrophosphate, 10% glycerol, 1% Triton X-100 and protease inhibitor cocktail (Complete Mini, Roche, Basel, Switzerland). For extracellular matrix isolation, cultured cells were treated with the hypotonic buffer NH_4_OH 20 mM for 20 min. After two washes with Phosphate-buffered saline (PBS) 1X the extracellular matrix present on the plastic plate was recovered with heated loading buffer (Laemmli solution) with the help of a scraper. Protein levels in cell-derived extracellular matrix were normalized with respect to the number of cells seeded and grown in the same conditions of cells used for extracellular matrix recovering. Conditioned media (CMs) were processed with Amicon Ultra-15 Centrifugal Filter Unit with Ultracel-3 membrane (Merk Millipore, Billerica, MA, USA) for concentration of proteins with a molecular mass greater than 5 kDa, according to the manufacturer’s instructions. Concentration factors ranged from 30 to 40X.

### Proteins analysis techniques

Western Blot. Protein lysates (20 μg), a fixed volume of solubilized extracellular matrix (20 μl) or a fixed volume of concentrated CM (26 μl) were mixed with loading buffer under reducing conditions, heated for 5 min at 95 °C, loaded on 4–12% precast NuPage SDS-Bis-Tris gels (Life Technologies, Carlsbad, CA, USA). The proteins were then transferred to PVDF membranes (Merk Millipore), stained with Red Ponceau to check loading and membranes saturated for 1 h at room temperature in blocking solution (5% low-fat milk in TBS + 0.1% Tween-20) before probing with the appropriate antibodies. Blots were washed with TBS-0.5% Tween-20 and further incubated with horseradish peroxidase-conjugated secondary antibodies (GE Healthcare, Little Chalfont, UK) for 1 h at room temperature. Western blots were developed using the enhanced chemiluminescence method (GE Healthcare) according to the manufacturer’s instructions. Data were acquired and analyzed using Quantity One 4.6.6 software (Bio-Rad, Hercules, CA, USA). The following primary antibodies were used: COL11A1 (1:500 rabbit polyclonal, NBP1–55803, Novus Biologicals, Littleton, CO USA), COL10A1 (1:1000 rabbit polyclonal, LS-C157654, LSBio LifeSpan Biosciences, Seattle, WA USA), SPARC (5 μg/ml mouse IgG1, 33–5500 Invitrogen), and vinculin (1:1000 mouse monoclonal, hVIN-1 clone, Sigma Aldrich). Vinculin quantification, concentration factor and cell counts were used to normalize ECM proteins in cell lysates, CMs and extracellular matrix preparation, respectively.

Circulating proteins were measured by using commercially available ELISA kits (COL10A1 and COL11A1 LifeSpan Biosciences, Inc., SPARC R&D), according to manufacturer’s instruction. Duplicate measures were performed for each sample. Protein levels were expressed as OD value as measured by Microplate Reader Tecan Infinite® M1000.

### Statistical analysis

After matching, the analysis of the levels of ECM molecules was performed on a set of 57 lung cancer patients and 57 healthy controls for COL11A1 and COL10A1 and on a set of 90 lung cancer patients and 90 healthy controls for SPARC. Raw absorbance values were corrected by exploiting the values of the ELISA standards. The distributions of the absorbance values of ECM molecules in plasma samples of lung cancer patients and healthy controls were compared by using the Wilcoxon test. The association between each molecule levels and clinicopathological variables was investigated using the Wilcoxon test for categorical variables and the Spearman correlation coefficient for continuous variables. A univariable logistic regression model including the molecule was fitted for the comparison between patients and healthy controls, and the area under the ROC curve (AUC) was estimated as a measure of discriminative ability Sensitivity, specificity, positive predictive value (PPV) and negative predictive value (NPV) corresponding to the cutoff of the ROC maximizing the Youden Index, were extrapolated**.** AUC, sensitivity/specificity, PPV/NPV 95% confidence intervals were estimated using bootstrap procedure (Efron B. An Introduction to the Bootstrap. Chapman and Hall, New York, NY; 1993). Multivariable quantile regression models with median as the reference quantile were implemented to study the association between the molecules and the disease status (tumor vs control), adjusting for possible confounders such as age, packyears, COPD and sex. Moreover, we studied the prognostic effect of ECM molecule levels on overall survival (OS). At univariable analyses each molecule was categorized according to its tertiles and the Kaplan-Meier curves were estimated and statistically compared by means of the log-rank test. Multivariable Cox models were also implemented, where the effect of the molecule was adjusted for age, packyears, COPD (present vs absent) and sex (M vs F); the corresponding Hazard Ratios (HR) were estimated. In all the models the molecules were included as continuous variables using 3-knots restricted cubic splines [33] The analyses were carried out using R software, version 3.2.0 (http://www.r-project.org/). The test results were considered statistically significant whenever a two-sided *p*-value below 0.05 was obtained.

## Results

### ECM-related gene expression profiles are enriched in lung cancer associated fibroblasts

We reasoned that novel potential circulating biomarkers derived from the tumor microenvironment could be developed starting from the discovery of biological pathways defining activated stroma. To identify factors responsible for a proficient cross-talk between fibroblasts and cancer cells in lung tumors, we have previously characterized cancer-associated fibroblasts (CAF) and normal fibroblasts (NF) isolated from patients with lung cancer (*n* = 60) by gene expression profiles. Gene Set Enrichment Analysis (GSEA) was used to identify groups of genes whose expression was specifically altered in cancer associated fibroblasts [23]. Class comparison analysis identified ECM deposition and remodeling among the most enriched pathways in CAF as demonstrated by the up-regulation of two important collagen isoforms, COL11A1 and COL10A1**.** Interestingly these two proteins also belong to a gene signature with prognostic value so-called ECM3, originally derived by studying expression changes of extracellular matrix genes in a cohort of breast cancer clinical specimens [24]. The ECM3 signature consists of 58 genes encoding 43 structural ECM proteins we found that 11 out the 58 genes were significantly differentially expressed between CAF and NF, each contributing to an enrichment of the ECM3 signature in the activated fibroblasts phenotype (Table 1). To understand the potential of ECM3-related genes as circulating biomarkers, we have focused on three individual proteins, COL11A1 and COL10A1 that resulted the most differentially expressed genes in CAF, and SPARC which is also present in the ECM3 signature but not significantly overexpressed in CAF. The prioritization of these candidates was based on fibroblasts gene expression profiling data for COL11A1 and COL10A1 and on the existing literature on SPARC in lung tumorigenesis [27].

**Table 1:** Modulation of ECM3 genes in CAF vs NF comparison (microarray analysis)

Gene title	Fold change	*p*-value
COL11A1	*0.716*	**0.008**
COL10A1	*0.709*	**0.001**
COL1A1	*0.707*	**0.032**
COL5A1	*0.655*	**0.010**
COL5A3	*0.620*	**0.022**
COL15A1	*0.618*	**0.143**
COL8A1	*0.616*	**0.049**
COL16A1	*0.603*	**0.002**
TIMP3	*0.577*	**0.200**
BGN	*0.503*	**0.052**
MMP11	*0.475*	**0.107**
PCOLCE	*0.443*	**0.242**
FN1	*0.437*	**0.009**
THBS1	*0.391*	**0.083**
COL6A3	*0.367*	**0.200**
COL1A2	*0.349*	**0.037**
FBN1	*0.345*	**0.021**
COL5A2	*0.329*	**0.245**
DCN	*0.289*	**0.136**
LAMC1	*0.256*	**0.079**
COL3A1	*0.242*	**0.461**
LEPRE1	*0.239*	**0.081**
SGCD	*0.191*	**0.362**
SERPINH1	*0.182*	**0.278**
HSPG2	*0.181*	**0.021**
ADAMTS2	*0.176*	**0.102**
SPARC	*0.173*	**0.390**
COL6A1	*0.172*	**0.474**
COL18A1	*0.139*	**0.353**
LAMA4	*0.137*	**0.399**
LAMB1	*0.118*	**0.500**
NID2	*0.106*	**0.615**
EMILIN1	*0.093*	**0.563**
COMP	*0.049*	**0.892**
CDH11	*0.047*	**0.808**
EFEMP2	*0.042*	**0.624**
COL6A2	*0.012*	**0.942**
LAMA2	*0.012*	**0.960**
SPARCL1	*0.007*	**0.950**
THBS2	*-0.013*	**0.964**
SPON2	*-0.031*	**0.951**
ADAM12	*-0.038*	**0.622**
ITGB5	*-0.041*	**0.816**
ITGBL1	*-0.051*	**0.735**
SERPINF1	*-0.119*	**0.807**
MMP2	*-0.130*	**0.421**
MMP14	*-0.188*	**0.044**
ADAMTS5	*-0.252*	**0.411**
CTSK	*-0.266*	**0.511**
COL14A1	*-0.286*	**0.213**
ELN	*-0.326*	**0.284**
FLRT2	*-0.379*	**0.072**
FBLN1	*-0.405*	**0.487**
SPON1	*-0.748*	**0.066**
SLIT3	*-0.763*	**0.078**

Italic: Positive enrichment; Bold: Negative enrichment

### ECM-related proteins are enriched in CAF and selectively released in conditioned medium

**Fig. 1:**
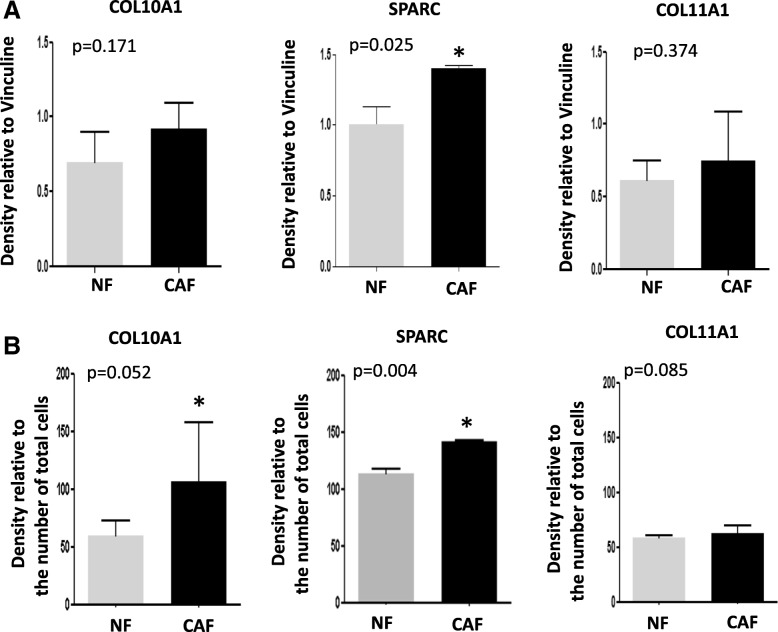
ECM related molecules are increased in CAF and selectively released in their conditioned medium: **a** Western Blot analysis of whole lysates of NF and CAF showing the protein levels of the three molecules (COL10A1, SPARC and COL11A1,) in CAF and NF cell cultures. The histograms show relative quantification by densitometric analysis normalized to Vinculin. **b** Western Blot analysis for the presence of the proteins COL10A1, SPARC and COL11A1 released in the CM of CAF or NF. The histograms show the densitometric analysis (in arbitrary Units) after normalization with respect to total number of cells. The difference was considered significant at *p*-value < 0.05

To validate the ECM-related genes expression patterns at protein level we performed western blot analyses on cell lysates of CAF (*n* = 3) and NF (n = 3) cell lines established from lung cancer patients and found that COL10A1 and SPARC were slightly enriched in CAF compared to NF (*p* = 0.171 and *p* = 0.025 respectively), while no differences in COL11A1 expression levels were detected (*p* = 0.374) (Fig. 1a).

To evaluate whether fibroblasts could represent the source of circulating ECM-related proteins and whether these factors are preferentially released in the medium conditioned by CAF, we analyzed by western blot conditioned medium obtained in serum-free conditions from CAF and NF (n = 3, respectively). The analysis revealed that CM by CAF was enriched in COL10A1 (*p* = 0.052) and SPARC (*p* = 0.004) as compared to NF, while only a trend in the enrichment of COL11A1 protein levels was found (*p* = 0.085) (Fig. 1b). We concluded that fibroblasts represent one possible source of the secreted proteins and that the differences in the release reflect their expression levels in cell cultures total lysates. To exclude protein deposition in a bound form in the extracellular matrix we also analyzed protein expression in the decellularized matrix without finding any of these proteins, thus indicating their preferential release.

### ECM-related proteins as lung cancer biomarkers

To demonstrate the potential clinical utility of ECM-related proteins as lung cancer circulating biomarkers, we analyzed by ELISA assay matched plasma samples from 57 patients (TU) and 57 controls (CTR) for COL11A1 and COL10A1 and from 90 patients (TU) and 90 controls (CTR) for SPARC. The matching was performed according to clinical parameters (sex, age, smoke).

**Fig. 2:**
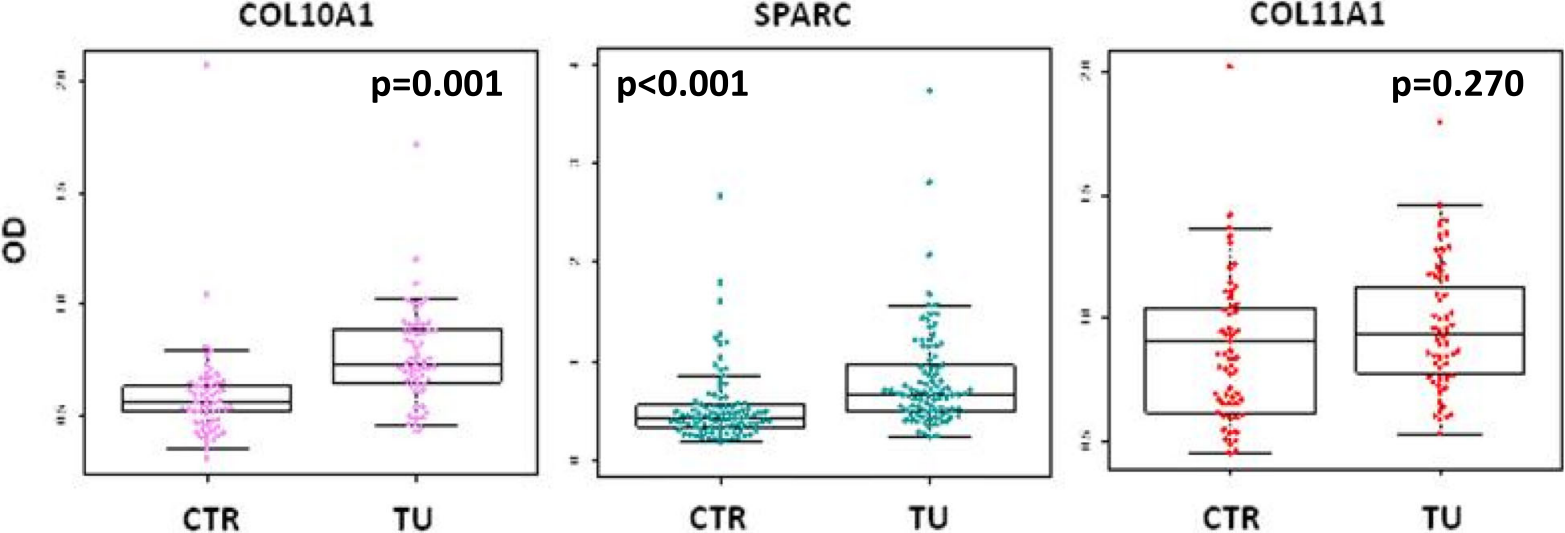
Levels of COL10A1 and SPARC are significantly higher in plasma of lung cancer patients compared to healthy heavy controls. **a** and **c** Box plots of COL10A1 and COL11A1 levels measured in plasma of 57 lung cancer patients (TU) and 57 healthy donors (CTR). **b** Boxplots of SPARC levels measured in plasma of 90 lung cancer patients (TU) and 90 controls (CTR), *p* = Wilcoxon test *p*-value

**Fig. 3:**
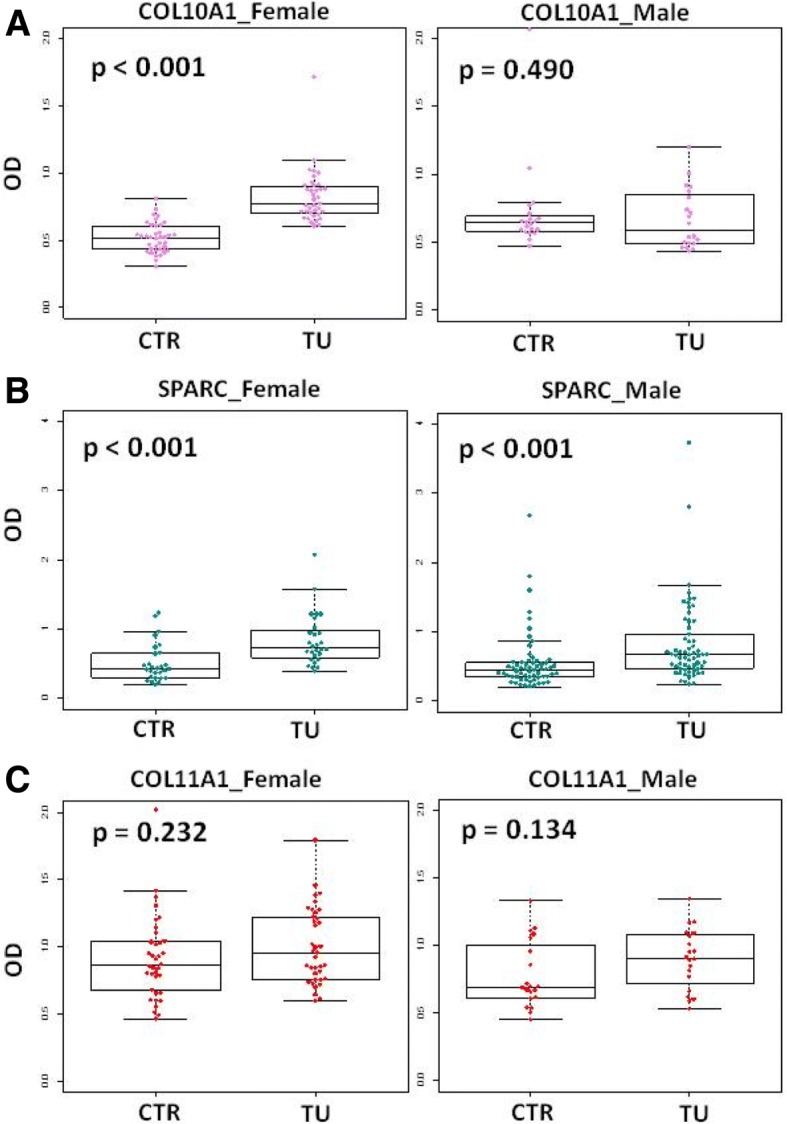
Circulating COL10A1 levels are preferentially increased in female lung cancer patients. Box plots showing levels of proteins in female and male groups of controls and patients separately (**a** = COL10A1, **b** = SPARC, **c** = COL11A1), *p* = Wilcoxon test *p*-value

Main characteristics of the subjects included in the present study are shown in Table 2. Overall, the results showed that the levels of COL10A1 and SPARC were significantly higher in lung cancer patients compared to healthy controls (COL10A1: *p* = 0.001; SPARC: *p* < 0.001), while no difference in the levels of COL11A1 could be detected (*p* = 0.270) (Fig. 2). We further explored the levels of the proteins in patients and controls stratifying by sex (COL10A1 and COL11A1: M = 20, F = 37, SPARC: M = 62, F = 28 for both groups). Interestingly we found different levels between patients and controls for COL10A1 only in the female subgroup (*p* < 0.001) while no differences were found in males (*p* = 0.490) (Fig. 3a). The difference in SPARC levels between patients and controls remained significant across gender subgroups (*p* < 0.001) (Fig. 3b) while there were no differences for COL11A1 in gender subgroups (*p* = 0.232) in female group and *p* = 0.134 in male group) (Fig. 3c). In multivariable quantile regression analysis, the COL11A1 protein levels resulted as associated with disease status (*p* < 0.001), while COL10A1 was no longer associated to the disease status (*p* = 0.814) (Additional file 1: Table S1). No significant association between OS and COL11A1 levels (*p* = 0.683) or COL10A1 levels (*p* = 0.960) was found (Additional file 2: Figure S1). Accordingly, the two variables resulted not significant at multivariable Cox analysis (HR = 0.79 [0.25–2.52] *p* = 0.923 for COL11A1 and HR = 1.06 [0.26–4.35] for COL10A1 *p* = 0.300).

**Table 2:** baseline characteristics of the subjects included in the study

Variables	COL11A1 COL10A1	COL11A1 COL10A1	SPARC	SPARC
	Healthy Controls (*n* = 57)	Patients (n = 57)	Healthy Controls (*n* = 90)	Patients (n = 90)
Sex (%)				
Male	20 (35%)	20 (35%)	62 (69%)	62 (69%)
Female	37 (65%)	37 (65%)	28 (31%)	28 (31%)
Age (mean,range)	66 (55–74)	65 (42–77)	66 (55–74)	66 (41–82)
Packyears (mean,range)			48 (20–112)	48 (4–158)
COPD (%)				
Present	22 (39%)	16 (28%)	48 (53%)	38 (42%)
Absent	35 (61%)	39 (68%)	42 (47%)	50 (56%)
NA		2 (4%)		2 (2%)
COPD severity (%)				
Severe	0 (0%)	0 (0%)	0 (0%)	2 (2%)
Moderate	5 (9%)	2 (3%)	4 (4%)	13 (14%)
Mild	17(30%)	14 (25%)	44 (49%)	23 (26%)
No	35 (61%)	39 (68%)	42 (47%)	50 (56%)
NA		2 (4%)		2 (2%)
Smoking history (%)				
Current	40 (70%)	21 (37%)	66 (73%)	64 (71%)
Former	17 (30%)	24 (42%)	24 (27%)	26 (29%)
Never	0 (0%)	12 (21%)		
Histology (%)				
Adenocarcinoma		47 (82%)		54 (60%)
Squamous		10 (18%)		22 (25%)
Others				12 (13%)
NA				2 (2%)
Tumor stage (%)				
IA		18 (31%)		20 (22%)
IB		3 (5%)		12 (13%)
IIA		9 (16%)		9 (10%)
IIB		4 (7%)		3 (3%)
IIIA		17 (30%)		28 (31%)
IIIB		1 (2%)		5 (6%)
IV		5 (9%)		6 (7%)
NA				7 (8%)
CT treatment (%)				
Yes		9 (16%)		13 (14%)
No		48 (84%)		77 (86%)
COL11A1 (median, IQR)	0.837 (0.653–1.043)	0.923 (0.759–1.172)		
COL10A1 (median, IQR)	0.556 (0.471–0.647)	0.739 (0.640–0.882)		
SPARC (median, IQR)			0.437 (0.343–0.555)	0.676 (0.501–0.956)

**Fig. 4:**
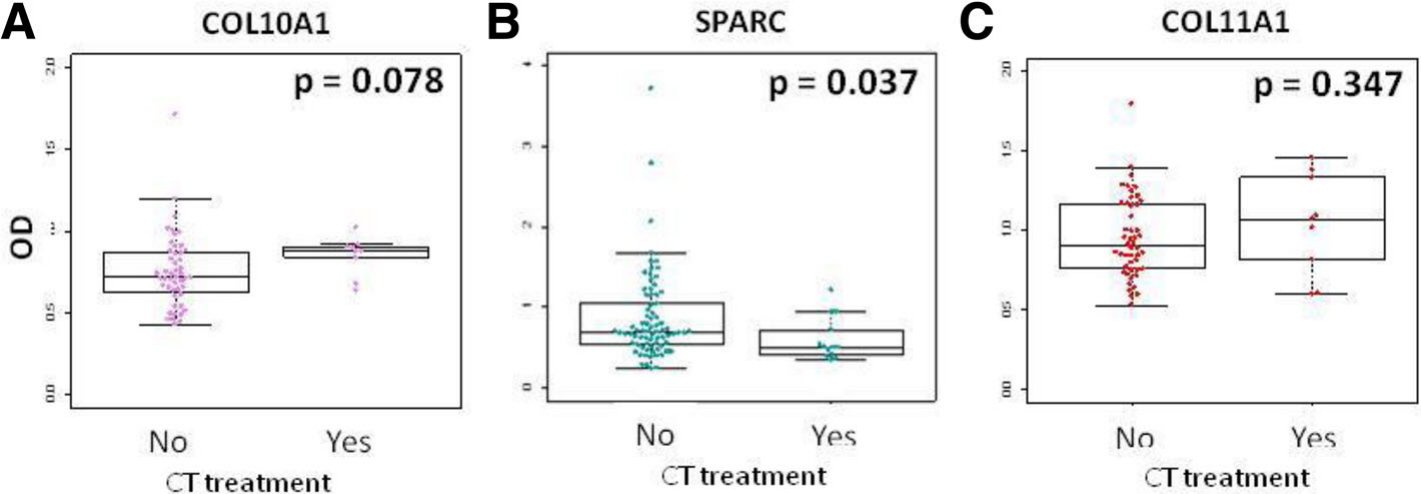
Circulating SPARC levels decrease in CT pretreated-lung cancer patients. Box plots showing ECM molecule (COL10A1, SPARC, COL11A1) levels after treatment with chemotherapy before surgery. *p* = Wilcoxon test *p*-value

Reflecting the clinical scenario of resected NSCLC a fraction of patients had received pre-operative chemotherapy (14–16% of patients depending on cohorts, see Table 2). To verify whether chemotherapy (CT) treatment affected circulating ECM molecule levels we considered patients that were treated before surgery and we found that the CT treatment was not associated with COL11A1 and COL10A1 levels (Fig. 4).

### Circulating SPARC is an independent diagnostic factor

Based on the inconsistent results on COL11A1 and COL10A1, we focused on SPARC protein. We performed univariable and multivariable analyses to assess the association between plasma SPARC levels and other established clinicopathological parameters such as age, smoking (in terms of packyears), tumor stage and COPD.

**Fig. 5:**
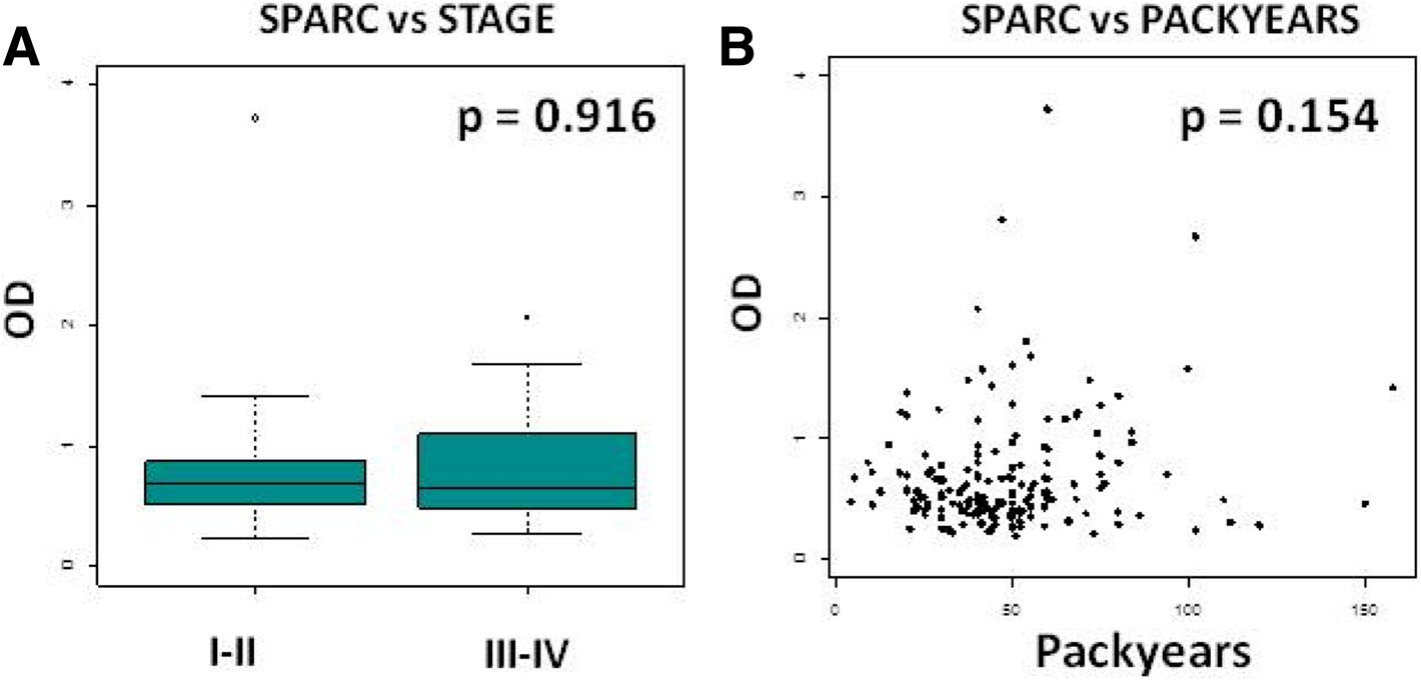
SPARC levels are not associated to tumor stage or smoking history (packyears). **a** Box plots showing SPARC levels in different tumor stages (I-II vs III-IV), *p* = Wilcoxon test *p*-value. **b** Correlation between SPARC levels and lifetime smoke exposure (packyears), *p* = Spearman correlation *p*-value

**Fig. 6:**
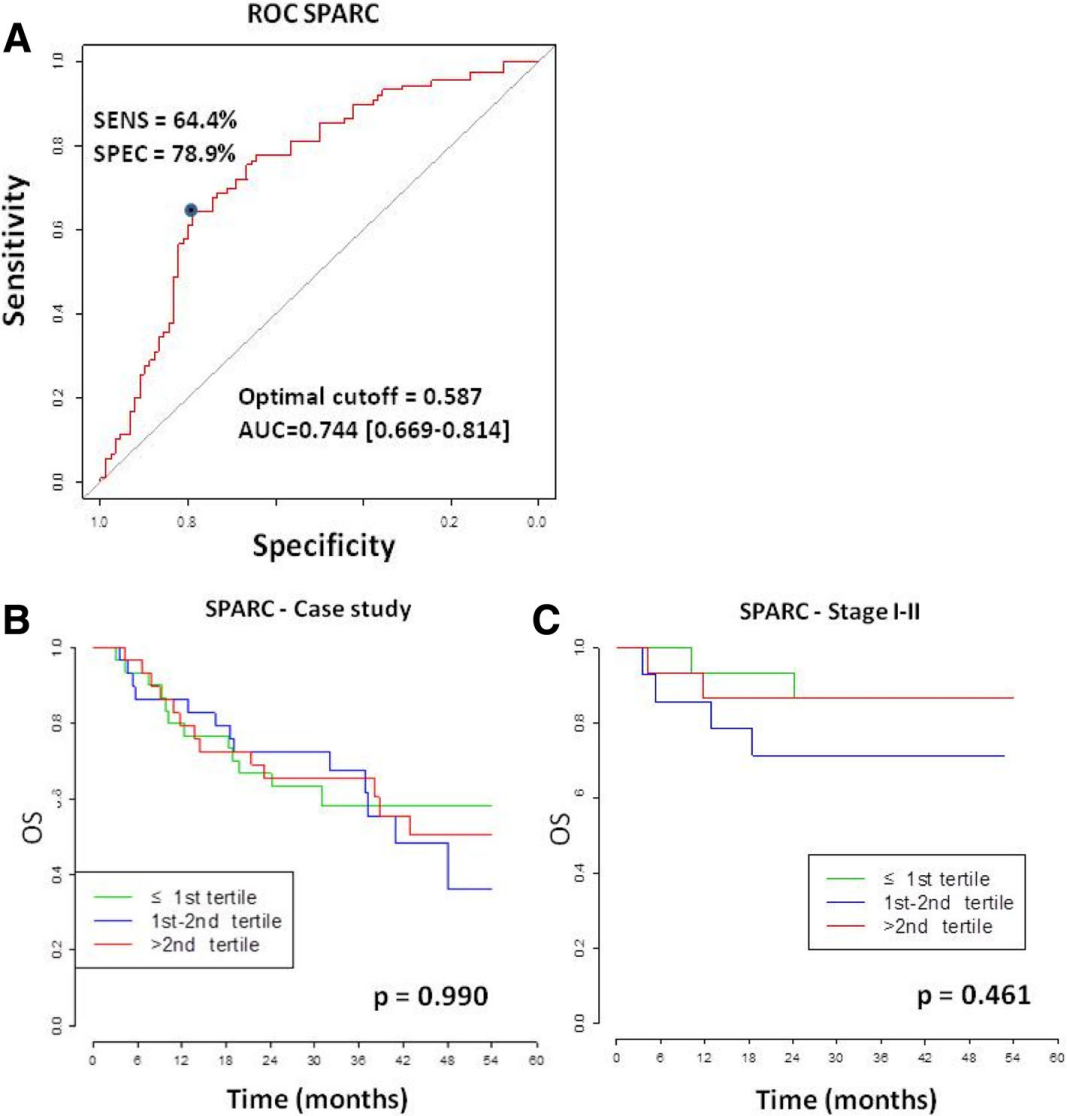
Circulating SPARC evaluation as a diagnostic and prognostic marker. **a** ROC curve of SPARC evaluating the capability of SPARC levels to discriminate between lung cancer patients and controls, with AUC and its bootstrap 95% CI. The optimal cutoff and sensitivity and specificity corresponding to the optimal cutoff of the ROC are reported. **b** Kaplan Meier overall survival (OS) curves for SPARC levels divided into three categories (≤1st tertile, 1st-2nd tertile, >2nd tertile) on the entire case series. **c** Kaplan Meier OS curves for SPARC levels divided into three categories (≤1st tertile, 1st-2nd tertile, >2nd tertile) on stage I-II patients, *p* = log-rank test *p*-value

As already assessed SPARC was significantly strongly elevated in lung cancer patients compared to healthy controls (*p* < 0.001) and remained significant across gender subgroups (*p* < 0.001). In univariable analysis, no association between SPARC protein levels and any of the clinicopathological characteristic was detected. No significant difference in SPARC levels was detected among tumor stages (*p* = 0.916), suggesting that circulating SPARC levels could represent a potential valuable biomarker in early stage lung cancer (Fig. 5a). Also, the correlation between SPARC levels and packyears resulted as not statistically significant (*p* = 0.154, Fig. 5b). In multivariable analysis, the protein levels were associated with disease status, even after adjustment for age, packyears, COPD and sex (*p* ≤ 0.001) (Table 3). Therefore, in both univariable and multivariable analyses, SPARC levels resulted higher in the presence of lung cancer. This is also mirrored by the ROC curve where circulating SPARC levels showed a good capability to discriminate patients from controls (AUC = 0.744, Fig. 6a). The optimal cutoff maximizing the Youden Index of the ROC for SPARC was 0.587 (corrected - OD values) corresponding to a sensitivity of 64.4% [54.4–74.4%] and specificity of 78.9% [70.0–87.8%] (Additional file 3: Table S3), and to a PPV of 73.7% [64.8–83.3%] and a NPV of 72.5% [65.1–83.1%]. Therefore, if SPARC is over the optimal cutoff, the probability of a true positive finding is 73.7%, while for values below the optimal cutoff the probability of a true negative is 72.5%.

**Table 3:** quantile regression multivariable analysis of association between SPARC levels and the characteristics of 90 lung cancer patients and 90 healthy controls

SPARC		
Variables	Difference between medians [CI]	*P* Value
Age (72 vs 62^a^)	0.002 [−0.003; 0.007]	0.563
Packyears (59 vs 33^a^)	0.001 [−0.001; 0.004]	0.647
COPD (Yes vs No)	−0.015 [−0.057; 0.055]	0.748
Sex (M vs F)	−0.046 [− 0.127; 0.035]	0.412
Disease status (Tumor vs Control)	0.255 [0.178; 0.285]	< 0.001

^a^the two values are, respectively, the 3rd and 1st quartile of the variable distribution

Concerning SPARC prognostic value, we did not find any association between OS and SPARC levels in tertiles both in the entire case series (*p* = 0.990) or considering early stage patients only (*p* = 0.461) (Fig. 6b, c). Also using the optimal cutoff for SPARC there was not any significant association (*p* = 0.971). Moreover, the molecule levels resulted not significant at multivariable Cox analysis, i.e. HR = 1.06 [0.57–1.99] (*p* = 0.593). Interestingly, SPARC levels were significantly lower for pre-treated patients (*p* = 0.037), indicating that SPARC levels could be an indicator of treatment response (Fig. 4).

## Discussion

In this study, we aimed to determine whether proteins related to the extracellular matrix are released in plasma and can potentially be useful as lung cancer biomarkers. We started our investigation from gene expression profiling of lung fibroblasts which are known to play an important role in cancer progression and ECM remodeling [20]. This approach revealed that ECM related proteins are particularly enriched in primary cultures of CAF from lung cancer patients [23] and also released in their conditioned culture medium. We then focused on molecules belonging to the ECM3 signature, previously identified in breast cancer and found to be prognostic when overexpressed in the most aggressive tumors [24]. We therefore analyzed two isoforms of collagens (COL11A1 and COL10A1 as the most expressed in lung fibroblasts gene profiling) and SPARC. Collagens represents the most abundant proteins of extracellular matrix and recent studies have highlighted an important role for COL11A1 and COL10A1 in many aspects of neoplastic progression [34]. Previous observations reported that COL11A1 is more expressed in CAF than in normal breast or pancreatic fibroblasts [35–37]. Overexpression of COL11A1 was also found in non-small cell lung cancer tissue samples where it correlates with pathological stage, presence of lymph node metastasis, and poor prognosis [36, 38]. Our study showed higher COL11A1 gene expression in CAF compared to NF, but no difference in protein expression in cells or its release in culture medium. In addition, we did not find any difference in protein levels in plasma of patient’s vs heavy-smokers controls highlighting the inadequacy of this protein as potential indicator of pathological features. This observation indicates that COL11A1 is albeit differentially expressed in CAF is not sufficiently discriminatory at the protein level.

Instead, COL10A expression showed remarkable significant difference between controls and lung cancer patients thus constituting a potential diagnostic candidate. However, subgroup analyses showed that this finding was restricted to the female group. In recent years, the importance of gender related biomarkers has gained more attention especially for lung cancer [39]. Several biological processes showed substantial differences between males and females in various hormonal states highlighting their impact on biomarker studies [40]. In this work even if we do not have additional data or insights to potentially explain such differences we underline that gender effects should be considered before starting any biomarkers development study. Our data suggest however that COL10A1 could represent a potentially promising biomarker for lung cancer in females and that its relevance could be potentially explored in gender-related cancers such as breast or ovarian or in specific subgroups in other cancers. The most promising results were obtained from the study on SPARC protein. Although SPARC has recently emerged as a prognostic biomarker in different tumors [41–43], its role in lung cancer remains controversial [44]. In non-small cell lung cancer, the localization (stromal or tumoral) of SPARC expression is associated with different disease prognosis. Absence of SPARC expression within the tumors is a negative prognostic factor [42] while high levels of SPARC protein expression, albeit rare, are associated with longer survival and could be protective against tumor aggressiveness [45]. On the other hand, patients bearing SPARC-positive stroma have significantly poorer overall survival [29]. Other studies found no prognostic impact of stromal SPARC expression [46]. To our knowledge, this is the first study showing the release of high levels of SPARC in plasma of lung cancer patients to indicate that this protein could represent a useful diagnostic biomarker for lung cancer. Based on the previous literature and given the detectable levels of SPARC in conditioned medium of fibroblasts, we can hypothesize that the source of circulating SPARC in our patients is from stromal fibroblasts, rather than from cancer cells, and that protein levels in plasma could reflect changes in a microenvironment that becomes activated and permissive to tumor growth and progression.

SPARC protein levels in plasma were high in all stages of the disease indicating that this could also serve as marker of early lung cancer. Consistently with a previous study [46] we did not find any association between levels of circulating SPARC and the prognosis of our patients, reinforcing the hypothesis that its presence reflects early changes in the microenvironment more related with initial tumor growth than relapse and metastasis. Therefore, we can speculate that SPARC expression could influence stroma responsiveness during tumor formation, representing an indicator of the disease at its earlier phases. The effect of SPARC on malignant progression may instead depend on other events such as EMT, growth factor or immune modulation [47, 48].

Most importantly, the multivariate analysis also confirmed the significant association between disease status and levels of circulating SPARC. Interestingly, the observation that SPARC could be a promising diagnostic biomarker was confirmed by the ROC analysis, showing an AUC of 0.744, with an optimal cutoff corresponding to 64.4% of sensitivity and 78.9% of specificity. However further studies involving larger numbers of subjects are required to confirm these results.

Interestingly, it has been suggested that ECM modification provides protection against chemotherapy-induced apoptosis and may play a role in the failure of cancer therapy [49]. Since the decrease (in terms of degradation) of ECM related proteins could be a marker of drug response we analyzed in this study patients that were treated with chemotherapy before surgery. Although our results are preliminary due to the low number of cases analyzed we report slightly decreased levels of SPARC protein in these patients indicating that beside a diagnostic marker SPARC may represent a marker of treatment response. Future longitudinal studies are however needed before firm conclusions can be drawn.

In conclusion, this is the first study to test circulating biomarkers related to ECM remodeling as possible diagnostic tools for lung cancer patients. SPARC emerged as the most promising biomarker, but it is possible that other genes identified in the comparative expression analysis of lung fibroblasts could be also useful for diagnostic purpose, either alone or in combination. It is now apparent, in fact, that panels of protein-based and nucleic acids-based cancer biomarkers, as opposed to single biomarkers, will probably be necessary for reliable cancer detection, especially to improve selection of high-risk individuals for CT screening and to distinguish malignant from benign nodules or identify patients with particularly aggressive cancers. Since the classical ELISA method that we used in our study suffer limitations in analysis time, sample size, equipment cost, and is not easily scalable to measure panels of proteins, new bioanalytical technologies should be developed to realize the full potential of protein biomarkers in the clinical setting. Our work represents an explorative study to verify whether proteins derived from ECM could be measured in plasma of lung cancer patients and their utility as circulating biomarkers and provides proof-of-concept on the feasibility and potential of this approach. Additionally, our study supports the concept that stroma-related plasma biomarkers may better fit as early diagnostic biomarkers than those strictly tumor-related. However, large prospective clinical studies are clearly warranted to confirm our preliminary results and further explore the existing potential of ECM-related circulating proteins.

## Conclusions

In conclusion, we show here that circulating extracellular matrix related protein could be used as potential biomarkers for early diagnosis of lung cancer. In particular, SPARC emerged as the most promising biomarker representing an indicator of the disease at its earlier phases. Interestingly also COL10A expression showed remarkable significant difference between controls and lung cancer patients thus constituting a potential diagnostic candidate. However, subgroup analyses showed that this finding was restricted to the female group. Our study also highlighted the inadequacy of COL11A1 protein as potential indicator of pathological features. Taken together, our findings sustain the hypothesis that stroma-related plasma biomarkers could represent promising early diagnostic biomarkers related to interactions between incipient tumors and the surrounding microenvironment.

## Additional files

Additional file 1: **Table S1.** Quantile regression multivariable analysis of association between COL11A1 and COL10A1 levels and the characteristics of 57 lung cancer patients and 57 healthy controls. (DOCX 15 kb)

Additional file 2: **Figure S1.** COL11A and COL10A levels are not associated with overall survival. Kaplan Meier overall survival (OS) curves for COL11A1 and COL10A1 levels divided into three categories (≤1st tertile, 1st-2nd tertile, >2nd tertile) on the entire case series *p* = log-rank test *p*-value. (PPTX 51 kb)

Additional file 3: **Table S2.** Sensitivity and specificity data for SPARC cutoffs. (DOCX 18 kb)

## Abbreviations

CAF: Cancer associated fibroblasts
CM: Conditioned medium
ECM: Extracellular matrix
GSEA: Gene Set Enrichment Analysis
NF: Normal fibroblasts
NSCLC: Non-small cell lung cancer
OS: Overall survival
SPARC: Secreted protein acidic and rich in cysteine
