# Supplementary material for: Diagnostic role of circulating extracellular matrix-related proteins in non-small cell lung cancer
Source: BMC Cancer. 2018 Sep 18;18:899. doi: 10.1186/s12885-018-4772-0 (PMC6145327; doi:10.1186/s12885-018-4772-0)
Supplement: Supplementary file 1 — Table S1. Quantile regression multivariable analysis of association between COL11A1 and COL10A1 levels and the characteristics of 57 lung cancer patients and 57 healthy controls. (DOCX 15 kb) [file 12885_2018_4772_MOESM1_ESM.docx]

**Additional file 1**

**Table S1**: quantile regression multivariable analysis of association between COL11A1 and COL10A1 levels and the characteristics of 57 lung cancer patients and 57 healthy controls.

| **COL11A1** | | |
| --- | --- | --- |
| **Variables** | **Difference between medians [CI]** | **P Value** |
| Age (72 vs 60^*^) | -0.001 [-0.013; 0.016] | 0.889 |
| Packyears (59 vs 32^*^) | 0.003 [0.000; 0.004] | 0.116 |
| COPD (Yes vs No) | -0.062 [-0.187; 0.050] | 0.436 |
| Sex (M vs F) | -0.151 [-0.302; 0.003] | 0.123 |
| Disease status (Tumor vs Control) | 0.253 [0.159;0.373] | **<0.001** |
| **COL10A1** | | |
| **Variables** | **Difference between medians [CI]** | **P Value** |
| Age (72 vs 60^*^) | -0.004 [-0.021; 0.022] | 0.682 |
| Packyears (59 vs 32^*^) | 0.001 [-0.003; 0.004] | 0.679 |
| COPD (Yes vs No) | 0.080 [-0.058; 0.337] | 0.524 |
| Sex (M vs F) | 0.324 [-0.117; 0.465] | 0.087 |
| Disease status (Tumor vs Control) | -0.022 [-0.164; 0.111] | 0.814 |

^*^the two values are, respectively, the 3^rd^ and 1^st^ quartile of the variable distribution
