# Supplementary figures and images for: Diagnostic role of circulating extracellular matrix-related proteins in non-small cell lung cancer
Source: BMC Cancer. 2018 Sep 18;18:899. doi: 10.1186/s12885-018-4772-0 (PMC6145327; doi:10.1186/s12885-018-4772-0)

## Slide 1
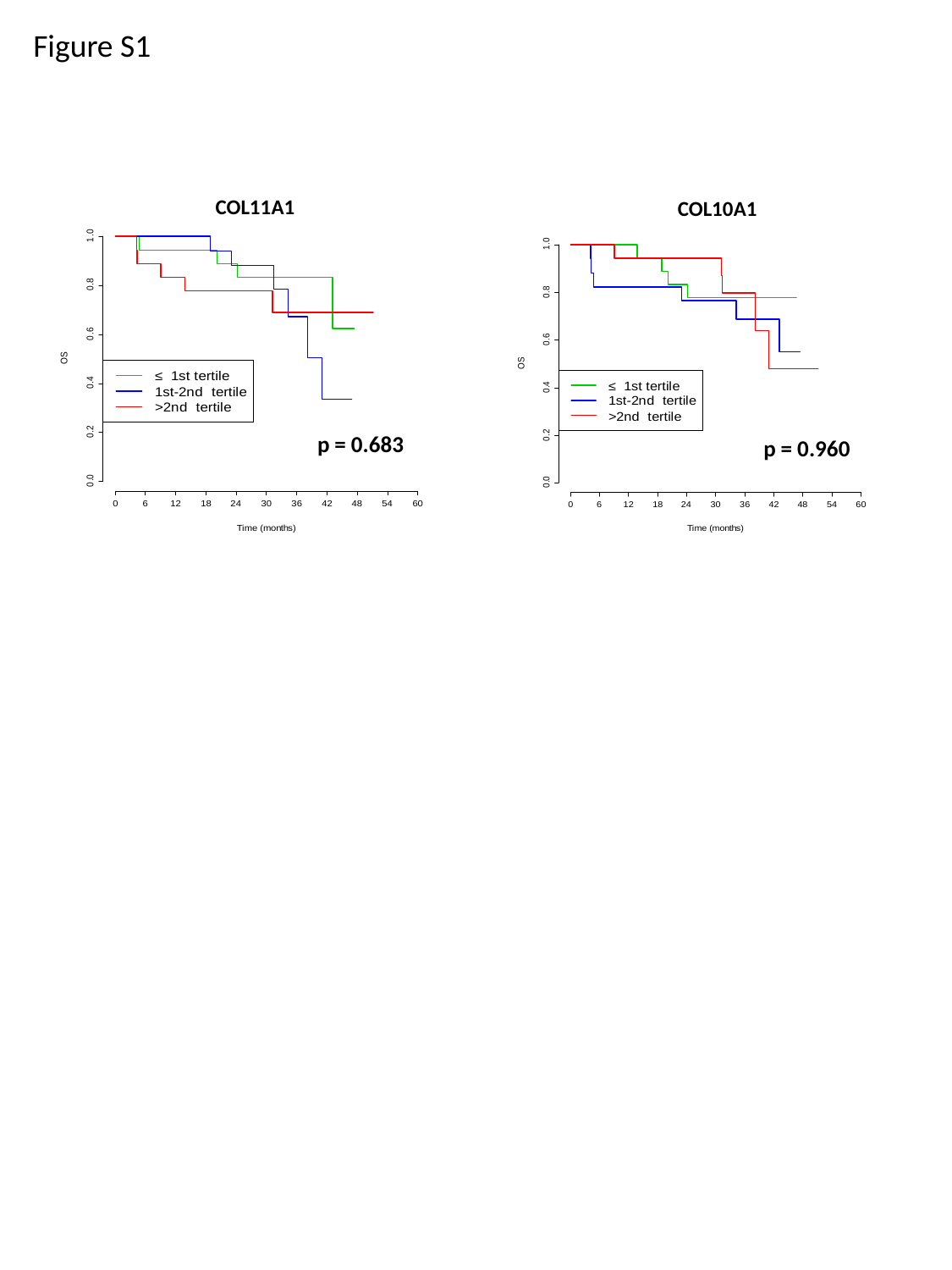

Figure S1
COL11A1
COL10A1
p = 0.683
p = 0.960

Supplement: Supplementary file 2 — Figure S1. COL11A and COL10A levels are not associated with overall survival. Kaplan Meier overall survival (OS) curves for COL11A1 and COL10A1 levels divided into three categories (≤1st tertile, 1st-2nd tertile, >2nd tertile) on the entire case series p = log-rank test p-value. (PPTX 51 kb) [file 12885_2018_4772_MOESM2_ESM.pptx]
