# Supplementary material for: Diagnostic role of circulating extracellular matrix-related proteins in non-small cell lung cancer
Source: BMC Cancer. 2018 Sep 18;18:899. doi: 10.1186/s12885-018-4772-0 (PMC6145327; doi:10.1186/s12885-018-4772-0)
Supplement: Supplementary file 3 — Table S2. Sensitivity and specificity data for SPARC cutoffs. (DOCX 18 kb) [file 12885_2018_4772_MOESM3_ESM.docx]

**Additional file 3:**

**Table S2**: sensitivity and specificity data for SPARC cutoffs.

| **Cutoffs** | **SENS**  **low** | **SENS**  **median** | **SENS**  **high** | **SPEC**  **low** | **SPEC**  **median** | **SPEC**  **high** |
| --- | --- | --- | --- | --- | --- | --- |
| 0.233 | 1.000 | 1.000 | 1.000 | 0.022 | 0.078 | 0.133 |
| 0.273 | 0.944 | 0.978 | 1.000 | 0.078 | 0.156 | 0.233 |
| 0.339 | 0.911 | 0.956 | 0.989 | 0.156 | 0.244 | 0.333 |
| 0.359 | 0.900 | 0.944 | 0.989 | 0.222 | 0.311 | 0.411 |
| 0.373 | 0.878 | 0.933 | 0.978 | 0.256 | 0.356 | 0.456 |
| 0.382 | 0.867 | 0.922 | 0.967 | 0.267 | 0.367 | 0.467 |
| 0.389 | 0.856 | 0.911 | 0.967 | 0.278 | 0.378 | 0.478 |
| 0.392 | 0.844 | 0.900 | 0.956 | 0.322 | 0.422 | 0.522 |
| 0.406 | 0.800 | 0.867 | 0.933 | 0.333 | 0.444 | 0.544 |
| 0.429 | 0.789 | 0.856 | 0.922 | 0.400 | 0.500 | 0.600 |
| 0.457 | 0.722 | 0.811 | 0.889 | 0.467 | 0.567 | 0.667 |
| 0.487 | 0.689 | 0.778 | 0.867 | 0.544 | 0.644 | 0.733 |
| 0.493 | 0.678 | 0.767 | 0.856 | 0.556 | 0.656 | 0.756 |
| 0.499 | 0.667 | 0.756 | 0.844 | 0.567 | 0.667 | 0.767 |
| 0.517 | 0.622 | 0.722 | 0.811 | 0.589 | 0.689 | 0.778 |
| 0.531 | 0.600 | 0.700 | 0.800 | 0.611 | 0.711 | 0.800 |
| 0.543 | 0.589 | 0.689 | 0.789 | 0.633 | 0.733 | 0.822 |
| 0.551 | 0.578 | 0.678 | 0.778 | 0.656 | 0.744 | 0.833 |
| **0.587^*^** | **0.544** | **0.644** | **0.744** | **0.700** | **0.789** | **0.878** |
| 0.621 | 0.511 | 0.611 | 0.711 | 0.711 | 0.800 | 0.878 |
| 0.643 | 0.478 | 0.578 | 0.678 | 0.722 | 0.811 | 0.889 |
| 0.656 | 0.467 | 0.567 | 0.667 | 0.744 | 0.822 | 0.900 |
| 0.677 | 0.400 | 0.500 | 0.600 | 0.756 | 0.833 | 0.911 |
| 0.738 | 0.289 | 0.389 | 0.489 | 0.767 | 0.844 | 0.911 |
| 0.767 | 0.267 | 0.367 | 0.467 | 0.789 | 0.856 | 0.922 |
| 0.799 | 0.233 | 0.333 | 0.433 | 0.800 | 0.867 | 0.933 |
| 0.859 | 0.222 | 0.311 | 0.411 | 0.811 | 0.878 | 0.944 |
| 0.916 | 0.200 | 0.289 | 0.389 | 0.822 | 0.889 | 0.944 |
| 0.933 | 0.189 | 0.278 | 0.367 | 0.833 | 0.900 | 0.956 |
| 0.989 | 0.156 | 0.244 | 0.333 | 0.844 | 0.911 | 0.967 |
| 1.043 | 0.144 | 0.233 | 0.322 | 0.866 | 0.922 | 0.967 |
| 1.182 | 0.111 | 0.189 | 0.267 | 0.878 | 0.933 | 0.978 |
| 1.200 | 0.100 | 0.178 | 0.256 | 0.889 | 0.944 | 0.989 |
| 1.248 | 0.078 | 0.144 | 0.211 | 0.911 | 0.956 | 0.989 |
| 1.308 | 0.067 | 0.133 | 0.200 | 0.922 | 0.967 | 1.000 |
| 1.634 | 0.011 | 0.044 | 0.089 | 0.944 | 0.978 | 1.000 |
| 1.936 | 0.000 | 0.033 | 0.078 | 0.967 | 0.989 | 1.000 |
| 2.735 | 0.000 | 0.022 | 0.056 | 1.000 | 1.000 | 1.000 |

^*^optimal cutoff; SENS = sensitivity; SPEC= specificity; low = lower bound of bootstrap 95% Confidence Interval; high = higher bound of bootstrap 95% Confidence Interval.
